# Exploring the Mechanism of *Panax notoginseng* Saponins against Alzheimer's Disease by Network Pharmacology and Experimental Validation

**DOI:** 10.1155/2021/5730812

**Published:** 2021-12-30

**Authors:** Yixuan Jiang, Shanliang Li, Xiaoqin Xie, Hemei Li, Panling Huang, Bocun Li, Lini Huo, Jing Zhong, Yuqing Li, Xing Xia

**Affiliations:** ^1^School of Pharmacy, Guangxi University of Chinese Medicine, Nanning 530200, China; ^2^Guilin People's Hospital, Guilin 541002, China; ^3^School of Basic Medical Sciences, Guangxi University of Chinese Medicine, Nanning 530200, China; ^4^School of Public Health and Management, Guangxi University of Chinese Medicine, Nanning 530200, China; ^5^Key Laboratory of TCM Pharmacology of Guangxi Education Department, Guangxi University of Chinese Medicine, Nanning 530200, China; ^6^Key Laboratory of Guangxi Zhuang and Yao Medicine, Nanning 530200, China

## Abstract

**Background:**

*Panax notoginseng* saponins (PNS) have been used for neurodegenerative disorders such as cerebral ischemia and Alzheimer's disease (AD). Although increasing evidences show the neuron protective effects of PNS, the vital compounds and their functional targets remain elusive. To explore the potential functional ingredients of PNS for the AD treatment and their molecular mechanisms, an *in vitro* neuron injured model induced by A*β* was investigated, and the potential mechanism was predicted by network pharmacology approach and validated by molecular biology methods.

**Methods:**

Network pharmacology approach was used to reveal the relationship between ingredient-target disease and function-pathway of PNS on the treatment of AD. The active ingredients of PNS were collected from TCMSP, PubChem database, and literature mining in PubMed database. DrugBank and GeneCards database were used to predict potential targets for AD. The STRING database was performed to reveal enrichment of these target proteins, protein-protein interactions, and related pathways. Networks were visualized by utilizing Cytoscape software. The enrichment analysis was performed by the DAVID database. Finally, neuroprotective effect and predictive mechanism of PNS were investigated in an *in vitro* AD model established by A*β*_25–35_-treated PC12 cells.

**Results:**

An ingredient-target disease and function-pathway network demonstrated that 38 active ingredients were derived from PNS modulated 364 common targets shared by PNS and AD. GO and KEGG analysis, further clustering analysis, showed that mTOR signaling targets were associated with the neuroprotective effects of PNS. In A*β*-treated PC12 cells, PNS treatment improved neuroprotective effect, including mTOR inhibition and autophagy activation.

**Conclusions:**

Collectively, the protective effects of PNS on AD-neuron injury are related to the inhibition of mTOR and autophagy activation.

## 1. Introduction

Alzheimer's disease (AD) is a progressive irreversible neurodegenerative disorder characterized by memory loss, cognitive impairment, and behavioral abnormalities. Neurons in the hippocampus of AD patients were destroyed gradually, leading to the loss of self-care ability in the later stage of the disease, which imposes a heavy burden on patients, carers, and families [1]. The aging of the world's population has brought about an increase in the number of AD patients, along with a growing medical burden. The WHO believes that AD should be considered a global public health priority [2, 3]. The main pathological characteristics of AD are amyloid *β*-protein (A*β*) deposition and neurofibrillary tangles (NFTs) [4]. A*β* deposition induces oxidative stress that interferes with cell signaling, causes hyperphosphorylation of tau proteins in the brain that damages nerve cells, and finally triggers neuroinflammation [5, 6]. Studies have shown that neurons in AD are protected when oxidative stress induced by A*β* can be ameliorated [7, 8]. Autophagy, a programmed death pathway that removes damaged proteins and organelles from cells, plays an important role in maintaining normal cellular function. Guo et al. proposed that a large number of autophagic vesicles were observed when neurons were damaged in the brains of AD animals, suggesting inducing autophagy is a potential mechanism for AD treatment [9]. Furthermore, rapamycin (an autophagy inducer) regulated PI3K/Akt1/mTOR/CREB pathway and thus reverses impaired redox homeostasis due to A*β* deposition, subsequently attenuated synaptic damage and increased neurotransmitters in AD rats [10].

*Panax notoginseng* (Burk) F. H. Chen is a famous traditional Chinese medicine (TCM) with a long history of use in activating blood circulation to dissipate blood stasis in China. *Panax notoginseng* saponins (PNS) are active ingredients extracted from *Panax notoginseng* (Burk) F. H. Chen. Recent studies had shown that PNS can enhance memory and cognition and also has a therapeutic effect on AD [11]. PNS is clinically used as the Xuesaitong injection, which is mainly used for neurological impairment caused by cerebral ischemia. There is a robust evidence that PNS possesses neuroprotective effect in AD; Huang et al. found that Xueshuantong not only reversed the reduction in cerebral blood flow but also significantly improved cognitive impairment, cleared A*β* deposits, and improved synaptic density in AD mice [12]. In another study, PNS ameliorated oxidative damage in early AD and exerted neuronal protective effects [13]. More interestingly, a recent study has been found that PNS also attenuate neurological damage in cerebral ischemia-induced neurological injury by regulating autophagy [14], but the evidence that autophagy is the key mechanism for protective effect of PNS on neurons in AD is still lacking. However, PNS are combination of multisaponin compounds that may act on multiple targets, and the etiology of AD is complicated; therefore, the specific targets and mechanisms underlying neuroprotection of PNS in AD have not yet been elucidated.

In recent years, network pharmacology has been widely used in the study of the mechanism of Chinese medicine. By predicting the possible targets and pathways of multicomponent Chinese medicines in complex diseases, computer-aided approaches showed important value in studying the mechanism of TCM. Based on the network pharmacology method, researchers have successfully examined the mechanism of active ingredients in some Chinese herbal medicines [15, 16].

PNS is herbal extract containing a variety of chemical components; its mechanism for protecting against neuronal injury in AD is still unclear. Whether the neuroprotective mechanism is related to autophagy and what are its targets need to be investigated thoroughly. Therefore, in this study, the potential targets and pathways of PNS were predicted by network pharmacology approaches, and the autophagy-related neuroprotective mechanism of PNS was validated in an *in vitro* AD model established by A*β*-induced damage to PC12 cells.

## 2. Materials and Methods

### 2.1. Screening of Functional Ingredients in PNS and Targets Prediction

The active ingredients in PNS were compiled from the Traditional Chinese Medicine Systems Pharmacology (TCMSP) database (https://old.tcmsp-e.com/tcmsp.php) and literature mining in the PubMed databank. Chemical structures were downloaded from PubChem (https://pubchem.ncbi.nlm.nih.gov/). The prediction of protein targets of active ingredients was using SwissTargetPrediction database (https://www.swisstargetprediction.ch/).

### 2.2. Screening of Protein Targets of Alzheimer's Disease

The potential targets for AD were collected from two resources: DrugBank database (https://go.drugbank.com/) and GeneCards database (https://www.genecards.org/). The results irrelevant to AD were manually removed and the nomenclature for remaining protein targets was standardized according to the Uniprot website (http://www.uniprot.org/).

### 2.3. Identification of Common Targets of PNS and AD

Venn diagram (http://bioinformatics.psb.ugent.be/webtools/Venn/) was used for identification of the common targets of PNS and AD.

### 2.4. Network Construction and Analysis

Protein-protein interaction (PPI) networks were constructed with STRING database (version 11.0, https://string-db.org/), and the network with confidence ≥0.7 was selected. Cytoscape software (version 3.7.2) was used for network construction and network analysis. Cluster analysis was performed by MCODE plugin of Cytoscape, and the two modules with the highest scores were obtained as the core target network for further analysis. GO functional annotation and KEGG analysis of the core target network were performed online by DAVID (https://david.ncifcrf.gov/).

### 2.5. Cell Culture and Treatment

PC12 cells were kindly supplied by Guangxi Key Laboratory of Efficacy Study on Chinese Materia Medica. Cells were maintained in DMEM supplemented with 10% FBS and incubated in a humidified atmosphere with 5% CO_2_ at 37°C. Based on different treatment, cells were divided into 6 groups: control group, model group, chloroquine group (CQ), PNS 1000 *μ*g/L group, PNS 500 *μ*g/L group, and PNS 100 *μ*g/L dose group. The control group was cultured with standard medium. The model group was treated with A*β* at concentration of 20 *μ*M (Sigma-Aldrich, USA, A*β*_25–35_ was dissolved in saline and aged in the incubator at 37°C for 7 days before use) for 24 h to induce cell injury. The chloroquine group was cotreated with A*β* 20 *μ*M and chloroquine 30 *μ*M (Sigma-Aldrich, USA) for 24 h. For the PNS groups, cells were incubated with A*β* 20 *μ*M + 1000 *μ*g/L PNS, A*β* 20 *μ*M + 500 *μ*g/L PNS, and A*β* 20 *μ*M + 100 *μ*g/L PNS (Chengdu Must Bio-Technology Co., Ltd., China; main components: notoginsenoside R1 14.5%, ginsenoside Rg1 28.0%, and ginsenoside Rb1 27.7%) for 24 h.

### 2.6. Transmission Electron Microscopy

After 24 hours of treatment in 6-well plates, cells were washed twice with PBS, gently scraped and collected after centrifugation, and then fixed with 3% glutaraldehyde overnight at 4°C. 12 hours later, cells were washed 3 times with PBS and post-fixed in 1% buffered osmium tetroxide for 2 h at 4°C. Cell pellets were dehydrated in an increasing ethanol gradient and embedded in epoxy resin. Ultrathin sections were obtained using the Leica EM UC7 and counterstained with lead citrate. A Hitachi HT7800 electron microscope was used to observe the cellular ultrastructure.

### 2.7. RNA Extraction and Quantitative Real-Time PCR

Total RNA of the cells was extracted using Eastep® Super Total RNA Extraction Kit (Promega, Shanghai, China). Concentration and purity of total RNAs were determined by ultramicro spectrophotometer. Reverse transcription was performed according to RevertAid First Strand cDNA Synthesis Kit (Thermo, Shanghai, China). Real-time PCR amplification was performed using SYBR green method according to GoTaq® qPCR Master Mix (Promega, China), and the CT values of the target primers were measured. The relative expression level of the target genes was calculated using the relative quantitative method 2^−ΔΔct^ and normalized to the *β-*actin level. Primer sequences (Takara Biomedical Technology, Beijing, China) were as follows: *p62*-F: *AGCTGCTTGGCTGAGTGTTAC*; *p62*-R: *CAATTTCCTGAAGAATGTGGG*; *β*-*actin*-F: *CATCCGTAAAGACCTCTATGCCAAC*; *β-actin*-R: *ATGGAGCCACCGATCCACA*.

### 2.8. Western Blotting Analysis

Total cellular proteins were extracted with Whole Cell Lysis Assay (KeyGEN Biotech, Nanjing, China); the protein concentrations were assessed using the bicinchoninic acid (BCA) method. The proteins were mixed with loading buffer and boiled for 5 min before subjected to SDS-PAGE electrophoresis. Each lane was loaded with 20 *μ*g cell lysates and separated by SDS-PAGE and then electro-transferred onto a polyvinylidene fluoride (PVDF) membrane (Millipore, USA). After blocked at room temperature for 2 h with 5% skimmed milk powder, the membranes were incubated overnight at 4°C with primary antibodies of LC3A/B (#12741s; 1 : 800), *β*-actin (#8457; 1 : 1000), mTOR (#2983T; 1 : 1000), and phospho-mTOR (#5536S; 1 : 1000), purchased from Cell Signaling Technology company and p62 (#18420-1-AP; 1 : 1000) from ProteinTech Group. 12 h after primary antibody incubation, the membranes were washed and incubated with a horseradish peroxidase-conjugated secondary antibody (BA1056; 1 : 10000; Boster Biological Technology) at room temperature for 2 h. The specific protein bands were visualized using an ECL kit, and band densities were quantified using Image J software.

### 2.9. Statistical Analysis

All quantitative data are presented as mean ± SD, SPSS 19.0 software was used for statistical analyze with one-way ANOVA for comparison between groups, and *P* < 0.05 was considered statistically significant.

## 3. Results

### 3.1. Compound-Disease Targets Network Construction

**Figure 1:**
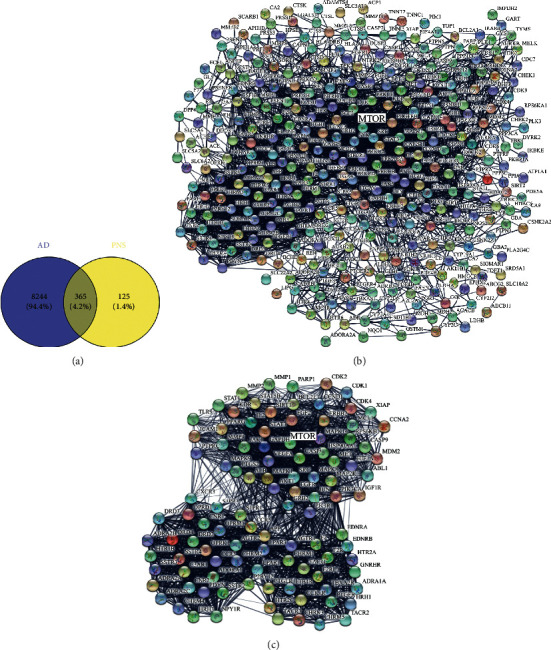
Network of compound-disease target interactions. Venn diagram (a) of the number of related targets shared by PNS and AD. PPI network (b) of the common targets shared by PNS and AD. PPI network (c) of the clustered core targets of PNS and AD.

A total of 38 active ingredients of PNS were obtained by using the following databases: TCMSP, China Knowledge Network, and PubMed. The active compounds included such as ginsenoside Rb1 and ginsenoside Rh2 of the protopanaxadiol-type saponins, ginsenoside Rg1 and ginsenoside Rh1 of the protopanaxatriol-type saponins, and ginsenoside R1 and ginsenoside R2 of oleanolic acid type saponins. Among these ingredients, 490 potential targets were collected by SwissTargetPrediction platform. Using “Alzheimer's disease” or “AD” as keywords, 8609 AD-related targets were obtained from the DrugBank and GeneCards databases. There are 365 common targets shared by PNS active ingredients and AD, and the ingredients-disease common target network is shown in Figure 1(a).

A network of 365 common targets connected by 6228 edges (Figure 1(b)) was obtained by analyzing the common targets of compound-disease via STRING database. The mean number of nodes in the network was 34.2, and the average local clustering coefficient was 0.497. To determine critical subnetworks from the common targets, MCODE in Cytoscape was used for cluster analysis, and two highest score modules containing a total of 105 core targets were obtained. The first module scored 37.708, containing 49 nodes and 905 edges. The second module scored 32.509, containing 56 nodes and 894 edges. The 105 core targets obtained from the merging of the two highest score modules were input into the STRING database to predict potential protein interaction network and to construct its topology network (Figure 1(c)).

**Figure 2:**
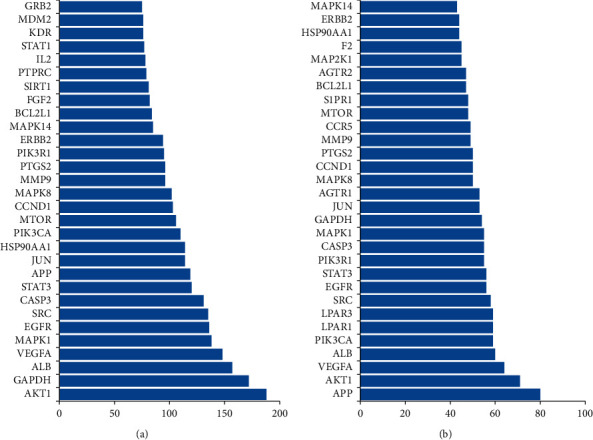
Results of target topological analysis. Degrees of the compound-disease common targets before clustering (a). Degrees of the core targets of the two highest score modules clustered by MCODE in Cytoscape (b).

**Figure 3:**
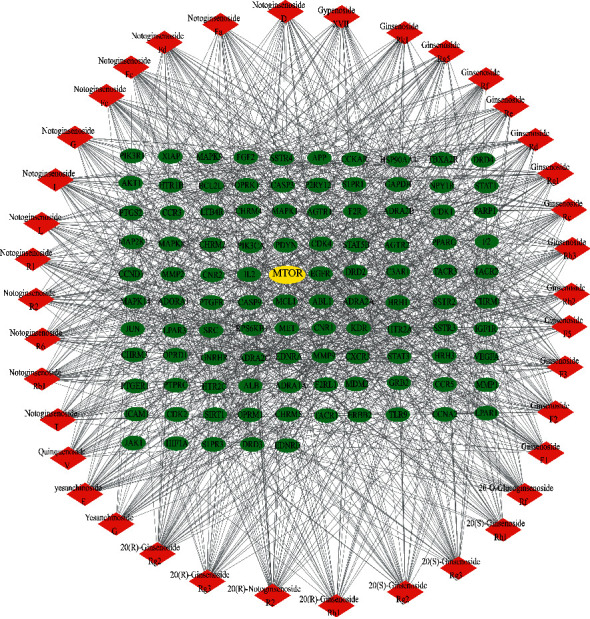
PNS ingredients-AD core targets network. The red nodes represent ingredients of PNS, the green nodes represent the clustered core targets of PNS and AD, and the yellow node represents the MTOR.

Network analyzer in Cytoscape was used for network topological characteristic analysis of the common targets (Figure 2(a)) or the core targets (Figure 2(b)). According to the degree of topological analysis, it showed that APP, AKT1, VEGFA, ALB, PIK3CA, LPAR1, LPAR3, SRC, EGFR, STAT3, PIK3R1, CASP3, MAPK1, and MTOR play important roles in the therapeutic effect of PNS in AD. Among them, molecular complex detection tool suggested that APP, AKT1, VEGFA, PIK3CA, EGFR, STAT3, and MTOR are potential core targets, because these network nodes whose eigenvalues were greater than the median were selected, which were also called key notes. The PNS ingredients-AD core targets network was drawn and visualized by Cytoscape in Figure 3. Among them, the core targets such as VEGFA, PIK3CA, and mTOR are not only related to most of the active ingredients of PNS but also have a close association with the main active ingredients of PNS.

### 3.2. GO Functional and KEGG Pathway Enrichment Analysis

Clue GO in Cytoscape was used to analyze the GO functional of the core targets. According to the results, a total of 108 significant GO terms were revealed (FDR < 0.05). The biological processes of these GO terms included G-protein-coupled peptide receptor activity, peptide receptor activity, neurotransmitter receptor activity, protein serine/threonine kinase activity, and phosphoprotein binding (Table 1). The core targets are mainly located at cell organelles including nucleoplasm, mitochondrial outer membrane, and cytosol. Related biological processes of target genes involved in signal transduction, cell communication, etc. The molecular functions of GO terms are involved in double-stranded DNA binding, ion binding, and heterocyclic compound binding. A total of 137 KEGG pathways were significantly enriched based on the core target of compound-disease network. The most significant 20 KEGG pathways are displayed in Table 2, such as neuroactive ligand-receptor interaction, PI3K-Akt signaling pathway, endocrine resistance, and EGFR tyrosine kinase inhibitor resistance.

**Figure 4:**
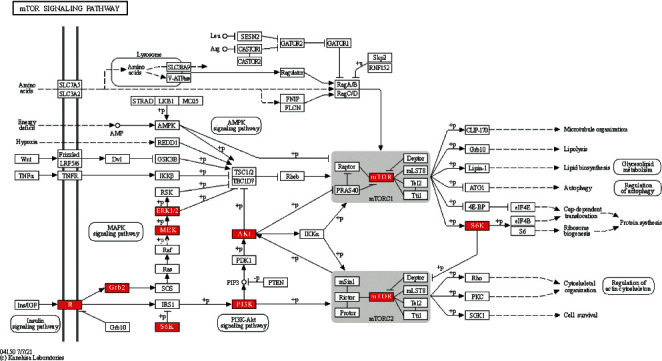
Detailed signaling pathway of mTOR receptor. Targets identified from the 108 common targets were highlighted in red.

**Figure 5:**
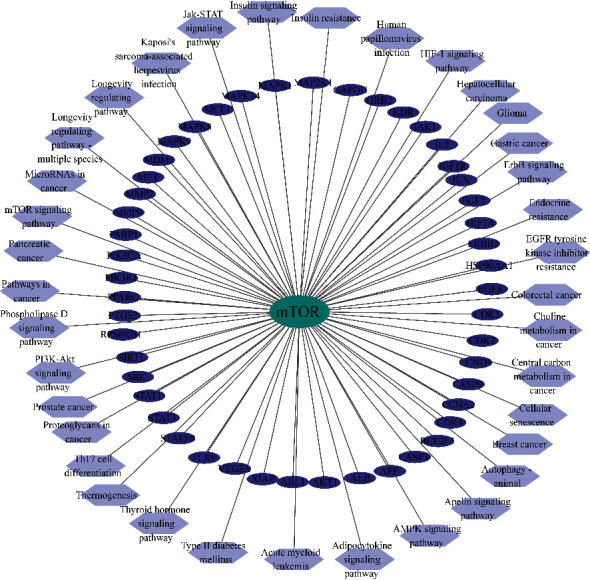
Target-pathway network of mTOR. The dark blue nodes represent the clustered core targets; the light blue nodes represent KEGG pathway.

mTOR is one of the core targets in compound-disease network. Not only does the mTOR pathway rank high, mTOR is also involved in many other important pathways, such as PI3K/Akt pathway, which is ranked third in Table 2, and mediates cell survival and proliferation mainly through mTOR.  Figure 4 shows the network diagram of mTOR with other core targets and pathways. This network diagram suggests that mTOR is involved in immunosuppression and regulates protein synthesis, etc. Moreover, mTOR regulates cell growth, apoptosis, autophagy, etc. It interacts with targets involved in cellular senescence and autophagy pathways such as Akt1, CCNA2, BCL2L1, and HIF1A. The correlation of mTOR with other core targets is visualized as mTOR-core targets pathways diagram in Figure 5. It indicated that PNS participates in immune suppression and protein synthesis and regulates cell growth, apoptosis, and autophagy. mTOR also interacts with key regulatory targets of cellular senescence and autophagy, such as Akt1, CCNA2, BCL2L1, and HIF1A. mTOR plays a vital role in AD pathology by controlling the programmed cell death pathways related to apoptosis and autophagy [17]. The results of the above network pharmacological analysis suggest that mTOR-related signaling pathway may be involved in the mechanisms of therapeutic effect of PNS in AD.

### 3.3. PNS Inhibited mTOR Activation in A*β*-Treated PC12 Cells

**Figure 6:**
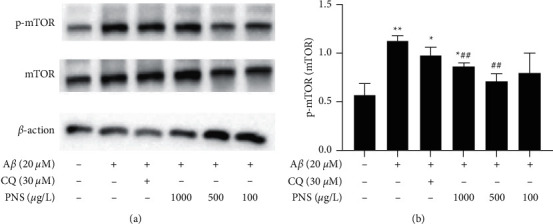
PNS inhibited mTOR activation in A*β*-treated PC12 cells. Note: values are expressed as the mean ± SD, *n* = 3. ^*∗*^*P* < 0.01 vs. control; ^*∗∗*^*P* < 0.01 vs. control; ^#^*P* < 0.05 vs. model; ^##^*P* < 0.01 vs. model.

To investigate the effect of PNS on mTOR pathway, we examined the level of p-mTOR and mTOR protein in A*β*-treated PC12 cells. As shown in Figure 6, the ratio of p-mTOR/mTOR was significantly increased in the PC12 cells treated with A*β*, when compared with normal cells (*P* < 0.01). Chloroquine, an autophagy inhibitor, tended to decrease the ratio of p-mTOR/mTOR, but not significantly (*P* > 0.05, compared with model group). Meanwhile, p-mTOR/mTOR ratios were decreased significantly after PNS 1000 *μ*g/L and 500 *μ*g/L treatment compared with the model group (*P* < 0.01).

### 3.4. PNS Enhanced Autophagy in A*β*-Treated PC12 Cells

**Figure 7:**
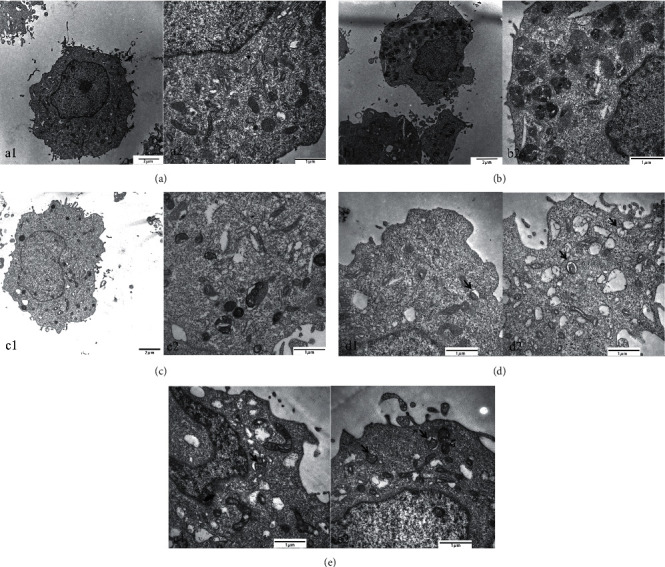
PNS activated autophagy in A*β*-treated PC12 cells. (a) Control group ((A): 10000X, (B): 30000X); (b) model group ((A): 10000X, (B): 30000X); (c) chloroquine group ((A): 10000X, (B): 30000X), (d) PNS 500 *μ*g/L group ((A), (B): 30000X); (e): PNS 100 *μ*g/L group ((A), (B): 30000X). Note: the arrows indicate autophagosomes.

To explore the level of autophagy in PC12 cells, cellular ultrastructure was observed using transmission electron microscopy. As shown in Figure 7, A*β*-induced dramatic changes on cell morphology in model cells including swollen and vacuolated mitochondria, but the autophagosome was not observed. Changes in cell morphology were also found in the chloroquine treatment group. Swollen and vacuolated mitochondria were more severe in it than in the model group. PNS treatment improved cell morphology damaged by A*β*. Swollen and vacuolated mitochondria are reduced autophagosomes that can be seen in cells from PNS 1000 *μ*g/L group and PNS 100 *μ*g/L-treated groups. Even more, vacuolated mitochondria enveloped by autophagosomes-like vesicles can also be found. These results indicated that PNS could induce autophagosome production in the A*β*-treated PC12 cells.

### 3.5. Effect of PNS on Marker for Autophagy Proteins and Genes in A*β*-Damaged PC12 Cells

**Figure 8:**
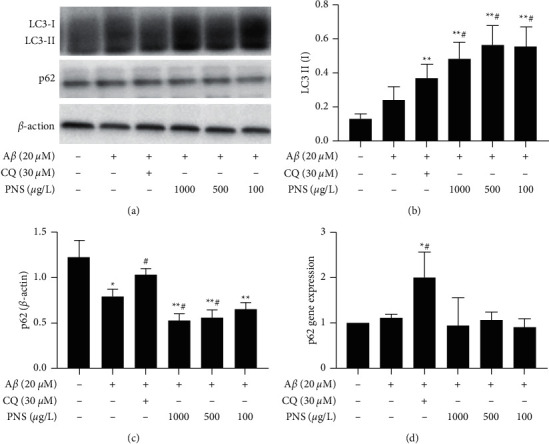
Effect of PNS on LC3II/I and p62 levels in A*β*-treated PC12 cells. (a) LC3-I, LC3-II, and p62 levels were detected by western blots. (b) Quantification of western blot data of LC3II/I, (c) p62 relative to *β*-actin, (d) *p62* mRNA level was determined by RT-qPCR with *β*-actin as control. Note: values are expressed as the mean ± SD, *n* = 3. ^*∗*^*P* < 0.05 vs. normal; ^*∗∗*^*P* < 0.01 vs. normal; ^#^*P* < 0.05 vs. model; ^##^*P* < 0.01 vs. model.

The levels of LC3II/I protein ratios and p62 proteins expression were determined by western blotting (Figures 8(a)–8(c)). A*β* induced an increase in LC3II/I ratio, as well as a decrease in p62 level significantly in model group (*P* < 0.05, compared with normal group). Chloroquine incubation had a trend to increase the ratio of LC3II/I without significance (*P* > 0.05, compared with model group), while it elevated p62 protein level significantly, compared with model group (*P* < 0.05). Interestingly, a remarkably increase of LC3II/I ratio was found after treatment of PNS; the ratio was even higher than that of the chloroquine group. In addition, p62 protein level was decreased significantly in PNS 1000 *μ*g/L group and PNS 500 *μ*g/L group (*P* < 0.05, compared with model group).

The mRNA expression of p62 was detected by RT-qPCR (Figure 8(d)). There was a trend to increase *p62* mRNA in A*β*-treated PC12 cells, after incubation with chloroquine; a significant elevation of *p62* mRNA expression was observed (*P* < 0.05, compared with model group). Meanwhile, no significant change of *p62* mRNA in PNS treated groups was found compared with model group (*P* > 0.05). The expression of *p62* mRNA had no significant effect under PNS treatment.

## 4. Discussion

The network pharmacology strategy is able to illustrate the complex interactions among the biological systems, drugs, and complex diseases from a network perspective and has been widely used in TCM research [18–21]. Network pharmacology method was used to systematically analyze the profile of multicomponent and multitarget of PNS and to explore the potential biomolecular networks of drugs in this research. Three main networks including compound-disease targets network, PNS ingredients-AD core targets network, and target-pathway network of mTOR were constructed and analyzed in this research. The first network uncovered the related targets between drug and disease. The second network explored the potential mechanism of PNS treating AD. The third network confirmed the feasibility of the second network. According to the analysis, the compounds with high degree were considered to be the core compounds of an herb and the pathways containing many targets linked by core compounds were decided to be genes regulated by the core compounds. In the present study, compound-disease targets network suggested that 38 active ingredients in PNS may interact with 365 targets which are related to AD. The result of target-disease-pathway network analysis showed that cytoplasmic membrane, PI3K-Akt pathway, autophagy, and cellular senescence pathway ranked at the top. The most important finding from the analysis is mTOR was ranked high and a large number of core targets were localized in or functionally linked to the mTOR pathway. It is reported that when mTOR signaling activation is impaired, microglial function has been impaired and autophagy regulation is also impaired, resulting in the increased incidence of AD [22]. mTOR can control the programmed cell death related to apoptosis and autophagy and plays a critical role in the pathology of AD. Inhibition of mTOR signaling will improve tau pathological conditions, and it has been reported that abnormal and sustained activation of PI3K/Akt/mTOR signaling is one of the early features of AD [23, 24]. Although some studies have found that PNS can improve learning and memory ability and has some neuroprotection effect in AD patients, the specific mechanism of action is still incomplete [12, 13]. Accordingly, we further explored the protective mechanism of PNS on AD-induced neurological injury. Hence, our network pharmacology results may support the hypothesis that PNS exerts therapeutic effect on AD through mTOR pathway while affecting cell survival, such as autophagy.

In the present study, we designed an *in vitro* experiment in PC2 cells to search for evidence for the hypothesis generated in network pharmacology study. One interesting finding is that treatment of PNS resulted in a decrease of p-mTOR/mTOR ratio which represents the reduction in the degree of mTOR activation. In accordance with the present results, Xue et al. have demonstrated that Xuesaitong injection, with PNS as the main component, significantly reduced p-mTOR and p-mTORC1 ratio, inhibited the activation of mTOR, and exerted antiapoptotic effect in podocytes of diabetic rats [25]. On the other hand, in spite of mTOR suppression, previous studies have provided evidence that PNS activated mTOR. Liu et al. [26] showed that PNS increased the level of p-mTOR, while inhibited Akt phosphorylation and changed the activity of the PI3K/Akt pathway in severe acute pancreatitis rats. Similarly, the increasing of p-mTOR, as well as activation of Akt/mTOR/PTEN pathway by ginsenoside Rb1, one of the main components of PNS, was reported in the focal cerebral ischemia rats [27]. These findings differ from the findings presented here. It is noteworthy that the basal extent of mTOR activation is decreased in both severe acute pancreatitis and cerebral ischemia. Therefore, it can be suggested that whether PNS activates or inhibits mTOR may depend on the basal extent of mTOR activation. The enhancement of PNS on mTOR activation in severe acute pancreatitis rats may partly be explained by the fact that the extent of mTOR activation was significantly reduced, rather than increased, and PNS may have a restore effect on mTOR activation extent. In the present study, the extent of mTOR activation was inhibited by treatment with PNS; this effect might result from that A*β* induced reduction in the extent of mTOR activation in PC12 cells. The effect of PNS on A*β*-treated PC12 cells is closely related to the change of mTOR activation; however, in-depth research would be required to elucidate the specific mechanisms underlying mTOR regulation of PNS in the nervous system of AD.

mTOR is closely related to aging, it is one of the key factors regulating autophagy, and inhibition of mTOR can enhance the level of cellular autophagy. The changes of mTOR activity remove damaged organelles via autophagy regulation; as a result, aging is delayed and neuron survival is improved [28, 29]. More recent attention has focused on the activation of mTOR pathway on autophagy; as a negative regulator of autophagy, the suppression of mTOR leads to autophagy activation, and the regulation of the mTOR pathway plays an important role in aging-related diseases [30]. One of the key pathological features of AD is A*β* protein deposition and neuronal fiber tangles. Apart from that, there is abnormal accumulation of autophagosomes in the degenerated neurons of the brain of AD patients, which may be caused by the dysfunction of autophagy regulation mechanism [31]. A*β*_25–35_ protein is well-known to be a toxic protein that causes neuronal damage in brain; it induces decline of neuronal activity, reduction of neuron number, and impairment of long-term memory, thus causes cognitive impairment [32]. A number of studies have revealed that activation of autophagy facilitated the phagocytosis of A*β* protein by autophagosomes and promoted autophagosomes fusion with lysosomes to form autophagolysosomes which leads to degradation of A*β*. It is now understood that autophagy has contributed to the clearance of neurotoxic proteins and damaged organelles in neurons and improved cognitive ability in AD [33, 34]. Inhibitions of the mTOR pathway by rapamycin, an mTOR inhibitor, decreased the level of p-mTOR significantly, reduced A*β* deposition, and delayed the progression of AD as a consequence of autophagy activation [35]. In a study which set out to determine the relationship of geniposide and autophagy, Zhang et al. found that geniposide, the active ingredient in *Gardenia jasminoides*, enhanced autophagy and increases the clearance of A*β* by inhibiting mTOR activity [36]. As discussed above, the activation of autophagy level in neurons caused by mTOR suppression has been found to be one of the key mechanisms of anti-AD agents. It is possible, therefore, that inhibition of mTOR by PNS may increase the level of autophagy, which may be one of the mechanisms underlying the therapeutic effect of PNS in AD.

PNS has been proved to be a highly effective therapeutic agent for AD. It can protect neuron against A*β*-induced injury and improve learning and memory ability in animal model of AD [13, 37]. In addition, some published studies have described the autophagy activation effect of PNS or the ingredients of PNS. In a study conducted by Liu et al., PNS has been found to protect against ischemia-reperfusion injury by activating autophagy in cardiomyocytes [14]. The same effect was also showed by Liang et al. that alleviation of cisplatin induced kidney injury in rats treated with PNS which was due to autophagy activation by modulating the HIF-1*α* pathway [38]. Moreover, ginsenoside Rb1, a main component of PNS, stabilized atherosclerotic plaques and reduced lipid accumulation in foam cells by promoting autophagy [39]. Notoginsenoside R1, known as another important component of PNS, also induced autophagy via activating PINK1, subsequently alleviated retinal vascular degeneration in diabetic retinopathy mice [40]. In the present study, A*β*_25–35_ was used on PC12 cells to induce an AD model in vitro; it was observed by transmission electron microscopy that PNS promoted the formation of autolysosomes and facilitated encapsulation of damaged mitochondria by autophagosomes in A*β*-treated PC12 cells. Meanwhile, western blot was used to detect the levels of autophagy marker proteins LC3-I, LC3-II, and p62; we demonstrated that LC3II/I ratio was significantly increased, while the p62 protein was reduced significantly after treated with PNS. However, at the transcriptional level, *p62* mRNA was not significantly altered by the treatment of PNS. LC3 and p62 are important proteins in autophagy; conversion of cytosolic form of LC3 (LC3-I) to membrane-associated form of LC3 (LC3-II) is a common indicator of autophagy [41]. The p62 protein is an important autophagy receptor that binds to LC3 to from a p62-positive envelope of LC3, and then degraded during autophagy. Thereby, it was found that autophagy inhibition is strongly correlated with the increase of p62 protein [42]. Furthermore, p62 also increases autophagosome by recruiting ubiquitinated substrates and promotes the conversion of LC3-I to LC3-II, enhancing the occurrence of autophagy [43]. As discussed above, the increase of LC3II/I and decrease of p62 indicated that PNS promoted autophagy, which corroborates our findings of transmission electron microscopy investigation. Likewise, in brain injury caused by cerebral ischemia, ginsenoside Rg1, a component of PNS, improved mitochondrial dysfunction and reduced brain injury by activating mitophagy [44]; it confirms, at least partly, our findings in the present study. The results obtained from the *in vitro* study in PC12 cells provide further support to the hypothesis presented in network pharmacology analysis: PNS suppresses the activation of mTOR which contributed to the promotion of autophagy and protects PC12 cells from A*β*-induced injury. There are lots of pathways or targets involved in the regulation of autophagy, including PI3K, Akt, Beclin-1, p52, and ULK autophagy regulatory proteins. However, what targets and pathways are possibly involved in the autophagy activation effect of PNS in AD warrant future in-depth investigation.

## 5. Conclusions

In summary, the present study has explored the mechanism underlying neuroprotective effect of PNS. The results of the network pharmacological analysis show that 38 active components in PNS may affect 364 potential targets related to AD, and mTOR could be one of the key targets of PNS. Our study *in vitro* has also shown that PNS treatment reduced activation of mTOR, which can contribute the autophagy activation observed in an AD-like neuron injury caused by A*β*_25–35_ in PC12 cells. Overall, the evidence from this study suggests that the protective effect of PNS on A*β* induced injury due to, at least part of, mTOR inhibition and autophagy activation. Despite its exploratory nature, this study certainly adds to our understanding of the neuroprotective mechanism of PNS. Thereby, on the basis of the present study, further studies regarding the specific mechanism and targets of PNS involved in the autophagy would be worthwhile.

## Figures and Tables

**Table 1:** Result of GO functional analysis.

Description	Count	FDR
G-protein-coupled amine receptor activity	17	8.17*E* − 25
G-protein-coupled peptide receptor activity	21	1.42*E* − 23
Peptide receptor activity	21	3.43*E* − 23
G-protein-coupled neurotransmitter receptor	12	6.82*E* − 18
G-protein-coupled serotonin receptor	11	4.78*E* − 17
Serotonin receptor activity	11	4.78*E* − 17
Neurotransmitter receptor activity	15	2.71*E* − 16
Neuropeptide receptor activity	9	1.32*E* − 11
Adrenergic receptor activity	6	4.43*E* − 10
Protein phosphatase binding	11	6.75*E* − 10
Phosphatase binding	12	9.97*E* − 10
Catecholamine binding	6	1.37*E* − 09
Acetylcholine receptor activity	6	3.51*E* − 09
G-protein alpha-subunit binding	6	4.66*E* − 09
Neuropeptide binding	6	4.66*E* − 09
G-protein-coupled receptor binding	13	1.12*E* − 08
Icosanoid receptor activity	5	1.91*E* − 08
Protein tyrosine kinase activity	9	1.02*E* − 07
Protein serine/threonine/tyrosine kinase activity	6	1.93*E* − 07
Ammonium ion binding	7	3.01*E* − 07
Prostanoid receptor activity	4	3.74*E* − 07
Insulin receptor substrate binding	4	3.74*E* − 07
Phosphatidylinositol phospholipase C	5	3.98*E* − 07
Phospholipase C activity	5	5.89*E* − 07
MAP kinase activity	4	1.12*E* − 06
Bioactive lipid receptor activity	4	1.12*E* − 06
Peptide binding	11	1.42*E* − 06
Hormone receptor binding	9	1.56*E* − 06
Protein serine/threonine kinase activity	13	1.98*E* − 06
Phosphoprotein binding	6	9.85*E* − 06

**Table 2:** Result of KEGG pathway enrichment analysis.

Term ID	Pathway name	Count	FDR
hsa04080	Neuroactive ligand-receptor interaction	48	6.66*E* − 56
hsa05200	Pathways in cancer	45	1.02*E* − 39
hsa04151	PI3K-Akt signaling pathway	30	2.71*E* − 25
hsa05167	Kaposi's sarcoma-associated herpesvirus infection	25	2.71*E* − 25
hsa01522	Endocrine resistance	21	3.65*E* − 25
hsa05212	Pancreatic cancer	19	7.79*E* − 24
hsa05205	Proteoglycans in cancer	24	1.34*E* − 23
hsa01521	EGFR tyrosine kinase inhibitor resistance	19	1.39*E* − 23
hsa04933	AGE-RAGE signaling pathway in diabetic complications	18	1.86*E* − 20
hsa04012	ErbB signaling pathway	16	1.63*E* − 18
hsa05206	MicroRNAs in cancer	18	1.27*E* − 17
hsa05215	Prostate cancer	16	1.29*E* − 17
hsa04068	FoxO signaling pathway	17	3.22*E* − 17
hsa05210	Colorectal cancer	15	5.56*E* − 17
hsa04917	Prolactin signaling pathway	14	1.27*E* − 16
hsa05203	Viral carcinogenesis	18	2.49*E* − 16
hsa04066	HIF-1 signaling pathway	14	7.61*E* − 15
hsa04024	cAMP signaling pathway	17	1.00*E* − 14
hsa05224	Breast cancer	15	5.98*E* − 14
hsa05214	Glioma	12	8.70*E* − 14
hsa04914	Progesterone-mediated oocyte maturation	13	1.03*E* − 13
hsa05165	Human papillomavirus infection	19	1.03*E* − 13
hsa05218	Melanoma	12	1.44*E* − 13
hsa05231	Choline metabolism in cancer	13	1.57*E* − 13
hsa04659	Th17 cell differentiation	13	2.42*E* − 13
hsa05220	Chronic myeloid leukemia	12	2.42*E* − 13
hsa05418	Fluid shear stress and atherosclerosis	14	2.56*E* − 13
hsa05226	Gastric cancer	14	7.94*E* − 13
hsa04014	Ras signaling pathway	16	1.06*E* − 12
hsa05221	Acute myeloid leukemia	11	1.34*E* − 12

## Data Availability

The datasets analyzed during the current study are available from the corresponding author on reasonable request. All materials used in this study are properly included in the Materials and Methods section.
